# Three-Year Outcomes After Temperature-Controlled Radiofrequency Ablation of the Posterior Nasal Nerve for Chronic Rhinitis

**DOI:** 10.1177/19458924251360889

**Published:** 2025-08-04

**Authors:** Jivianne T. Lee, Gregory M. Abbas, Daniel D. Charous, Mandy Cuevas, Önder Göktas, Patricia A. Loftus, Nathan E. Nachlas, Elina M. Toskala, Jeremy P. Watkins, Detlef Brehmer

**Affiliations:** 1 Department of Head and Neck Surgery, 21767 UCLA David Geffen School of Medicine, Los Angeles, CA, USA; 2 Advanced ENT and Allergy, Louisville, KY, USA; 3 Arizona Desert Ear, Nose & Throat Specialists, Goodyear, AZ, USA; 4 Department of Otorhinolaryngology Head and Neck Surgery, Faculty of Medicine and University Hospital Carl Gustav Carus, Technische Universität Dresden, Dresden, Germany; 5 ENT-Center, HNO-Zentrum am Kudamm, Berlin, Germany; 6 Department of Otolaryngology-Head and Neck Surgery, 8785 University of California, San Francisco, CA, USA; 7 ENT and Allergy Associates of Florida, Boca Raton, FL, USA; 8 Department of Otolaryngology – Head and Neck Surgery, 23217 Thomas Jefferson University, Philadelphia, PA, USA; 9 Fort Worth ENT Group, Fort Worth, TX, USA; 10 Faculty of Medicine, 12263 University Witten/Herdecke, Witten, Germany; 11 Department of Electrical Engineering and Applied Natural Sciences, 38932 Westphalian University of Applied Sciences, Gelsenkirchen, Germany; 12 Department of Otorhinolaryngology, Private ENT Practice, Göttingen, Germany

**Keywords:** chronic rhinitis, posterior nasal nerve, rTNSS, temperature-controlled radiofrequency, allergic rhinitis, vasomotor rhinitis, congestion, ablation, neurolysis, postnasal drip

## Abstract

**Background:**

Chronic rhinitis (CR) is characterized by refractory symptoms such as rhinorrhea, sneezing, nasal congestion, postnasal drip (PND), and cough. Most patients do not achieve lasting symptom relief with medical management.

**Objective:**

To evaluate the long-term efficacy and safety of temperature-controlled radiofrequency treatment targeting posterior nasal nerves (PNNs) for CR.

**Methods:**

This prospective, single-arm, open-label, multicenter study included patients aged 18–85 years across 19 centers in the United States and Germany. Outcome measures included reflective Total Nasal Symptom Score (rTNSS), PND and cough scores, and the Mini Rhinoconjunctivitis Quality of Life Questionnaire (MiniRQLQ). Outcomes, including adverse events, were reported through 3 years post-procedure.

**Setting:**

All procedures were performed in an outpatient office-based setting.

**Results:**

One hundred twenty-nine patients received treatment; 101 completed 3-year follow-up. The adjusted mean rTNSS Score improved from 7.8 (95% confidence interval [CI]: 7.5-8.1) at baseline to 3.2 (95% CI: 2.8-3.7) at 3 years (mean change: −4.5 [95% CI: −5.1 to −4.0]; *P* < .001). Rhinorrhea symptom scores improved from 2.6 to 1.2 (55.8% reduction). Compared to baseline, at 3 years, adjusted mean cough and PND scores declined from 1.3 to 0.4 (mean change: −0.9; *P* < .001, 69% reduction) and from 2.4 to 1.2 (mean change: −1.2; *P* < .001, 50% reduction), respectively. MiniRQLQ scores were significantly reduced from an adjusted mean of 3.0 (95% CI: 2.8-3.2) at baseline to 1.2 (95% CI: 1.0-1. 4) at 3-year follow-up; *P* < .001. No serious device- or procedure-related adverse events were reported.

**Conclusion:**

A single temperature-controlled radiofrequency treatment of the PNN safely and effectively reduced CR symptoms, including cough and PND, improved quality of life, and decreased medication burden through a period of 3 years with no serious adverse events.

## Introduction

Chronic rhinitis (CR) is characterized by inflammation of nasal mucosa for >12 weeks/year, resulting in rhinorrhea, sneezing, nasal congestion, itching, refractory postnasal drip (PND), and cough.^1–3^ Pharmacological treatments such as antihistamines, anticholinergic sprays, intranasal corticosteroids (INCS), and immunotherapy are first line of management for CR.^4^ However, many patients with CR do not have long-lasting relief from pharmacological treatments alone. Historically, when first-line medical treatment fails, CR is treated surgically.^5^

The posterior nasal nerves (PNNs) are branches of the sphenopalatine ganglion, which extend to the nasal cavity and surrounding structures. These nerves provide sensory innervation to the posterior regions of the nasal passages and sinuses.

Hyperactivity of these nerves results in excessive release of neuropeptides, resulting in neurogenic inflammation, contributing to refractory vasomotor and allergic CR symptoms.^6^ Nasal mucosal denervation by ablation of the PNNs reduces parasympathetic stimulation to the nasal mucosa, resulting in long-lasting relief of CR symptoms.^7^ Ablation of the nerves in the PNN region can be performed through surgical dissection, cryotherapy,^8^ laser,^9^ traditional radiofrequency,^10^ or minimally invasive temperature-controlled radiofrequency (TCRF).

The effectiveness and safety of TCRF PNN ablation for improving symptoms of CR have been previously reported in a single-arm pivotal study^11,12^ and a randomized sham-controlled trial.^13,14^ A previous report on this study demonstrated that TCRF ablation of PNNs using RhinAer provided a safe and effective treatment for CR, with symptom relief observed from 3 months^15^ through 2 years post-treatment.^16^ However, nerve regeneration can occur at a rate of 1 inch per month.^17,18^ Therefore, long-term data are useful to further evaluate the durability of the TCRF procedure for improving CR symptoms. In this study, we report the 3-year clinical outcomes.

## Methods

### Study Design

This prospective, open-label, single-arm, multicenter study was conducted between October 2020 and March 2024 at 16 centers in the United States and three centers in Germany. Patients were followed through 3 years post-treatment.

### Study Participants

This study included patients diagnosed with CR enrolled in the primary study following signed informed consent.^15^ Patients were evaluated in-office through 6 months and then remotely through 3 years post-treatment. The key inclusion and exclusion criteria have been previously reported^15^ and are detailed in Supplemental Table 1. Briefly, eligible participants were adults aged 18-85 years with a history of CR symptoms for at least 6 months and moderate to severe rhinorrhea and/or nasal congestion (rTNSS score ≥6). Exclusion criteria included anatomical obstructions limiting access to the posterior nasal passage, prior nasal surgery, active nasal or sinus infections, and conditions that could impair wound healing or compliance with study requirements.

### Treatment

Treatment was performed using the RhinAer^®^ System (Aerin Medical, Mountain View, USA) in an office setting. Investigators were board-certified otolaryngologists trained to use the TCRF device. After topical anesthesia, mucosa overlying the posterior middle meatus and superior portion of the posterior inferior turbinate in the region of the PNN was targeted for treatment. Treatment was administered with 1-5 nonoverlapping applications per side, treatment settings were temperature 6 °C, 4 W power, 12-s treatment time. No repeat (touch-up) treatment was allowed.

### Patients Assessments and Outcomes

The 24-h reflective Total Nasal Symptom Score (rTNSS) was recorded at baseline through 3 years. The primary efficacy endpoint of the study was the mean change in rTNSS at each follow-up compared to the baseline. Each symptom (nasal congestion, rhinorrhea, sneezing, and nasal itching) was scored on a 4-point scale (0-3), with higher scores indicating more severe symptoms. Additionally, a ≥30% improvement from baseline is considered a minimal clinically important difference (MCID) for determining treatment response, with treatment responders defined patients achieving this threshold at follow-up. The percentage of patients reporting each rTNSS subscore at baseline and each follow-up time point was determined. Self-reported symptoms of PND and cough were also recorded on a 4-point scale (0-3) scale at baseline and follow-up time points, consistent with prior studies.^15^ Patient quality of life (QoL) was assessed at baseline and each follow-up using the validated Mini Rhinoconjunctivitis Quality of Life Questionnaire (MiniRQLQ), a 14-item questionnaire that evaluates five symptom domains based on a 1-week recall period.^15^ Each item is scored on a 7-point scale (0-6), with higher scores indicating greater impairment. The MiniRQLQ MCID was defined as a ≥0.4-point improvement from baseline.

Medication use was not mandated by the study protocol; however, the use of various medication classes, including antihistamines, decongestants, oral leukotriene inhibitors, intranasal steroid sprays, and an intranasal anticholinergic spray was recorded at baseline and tracked through 3 years.

### Statistical Analysis

Statistical analysis was performed using SAS/STAT version 9.4 (SAS Institute, Cary, NC, USA). The study was powered to detect a mean difference in rTNSS from baseline to 3 months. Using an assumption of a mean difference of 1 point and a standard deviation of 3, 140 participants were required to provide 90% power, after accounting for a 15% attrition rate.

For the long-term follow-up study, general estimating equation models were used to evaluate repeated measures of continuous data (e.g., rTNSS and MiniRQLQ) over time. Adjusted (least squares) mean, mean change, % change, and 95% confidence interval (CI) are presented with *p*-values for changes between baseline and each follow-up visit. An independent *t*-test was used to evaluate differences in the demographics and characteristics of patients participating in the 3-year follow-up and those who did not. A linear regression model was used to assess the relationship between time (years) and the outcome measure from 1 to 3 years, assuming a continuous and linear trend in efficacy over time. Significance was accepted at a two-sided 0.05 alpha level.

## Results

### Demographics and Baseline Characteristics

**Figure 1.**
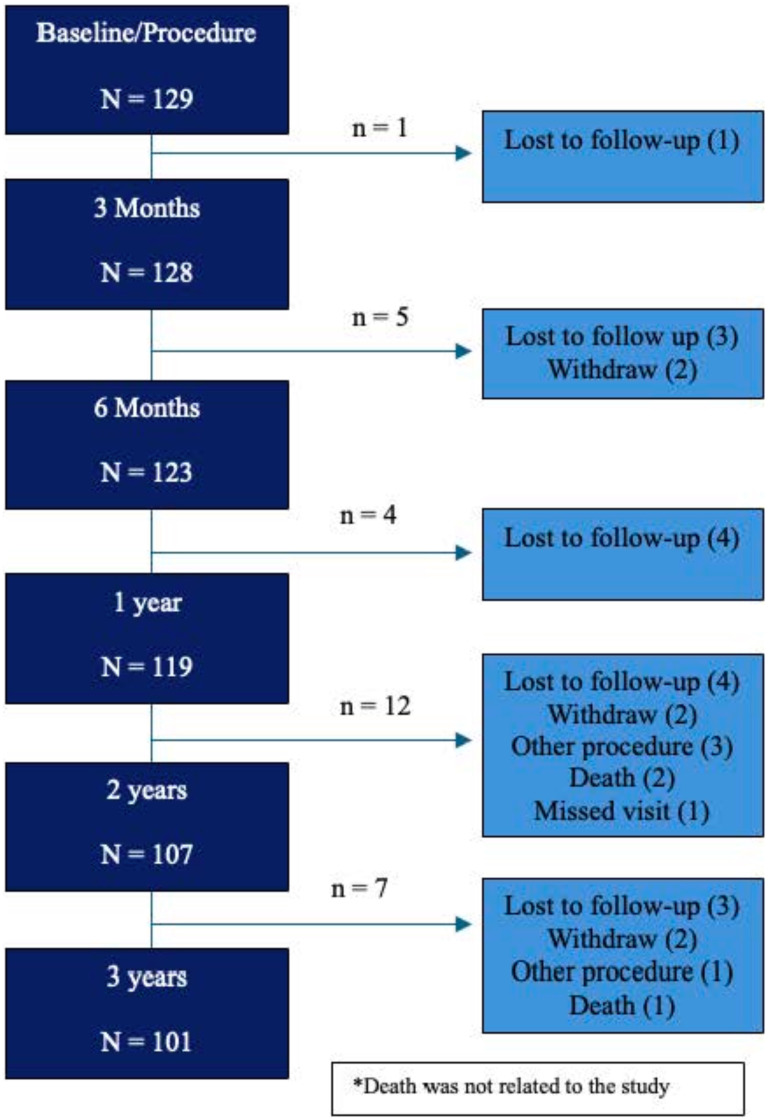
Patient Disposition.

A total of 129 eligible patients underwent treatment. Of the 129 patients, 101 patients were included in the analysis at 3 years post-procedure; 15 were lost to follow-up, six withdrew, three died (unrelated to the study), and four underwent additional nasal procedures (sinus surgery, inferior turbinate reduction, and cryoablation of the PNN) (Figure 1). There were no statistically significant differences in patient demographics or baseline characteristics between the enrolled population and those included in analysis at 3 years (Table 1).

**Table 1.** Patient Demographics and Baseline Characteristics.

Characteristic	Baseline (*N* = 129)	3-Year Follow-up (*N* = 101)	Exited Prior to 3 Years (*N* = 28)	*P*
Age, years, mean (SD)	57.8 (13.4)	58.0 (13.6)	57.2 (13.0)	.777
BMI, kg/m^2^, mean (SD)	27.2 (5.7)	27.2 (5.8)	26.9 (5.2)	.754
Sex, *n* (%)				
Female	69 (53%)	57 (56%)	12 (43%)	.202
Male	60 (47%)	44 (44%)	16 (57%)	
Race, *n* (%)				
Asian	4 (3%)	4 (4%)	0 (0%)	.576
White	117 (91%)	90 (89%)	27 (96%)	.461
Black/African American	5 (4%)	5 (5%)	0 (0%)	.585
Other	3 (2%)	2 (2%)	1 (4%)	.523
Nasal exam findings				
Turbinate enlargement	30 (23%)	23 (23%)	7 (25%)	.805
Nasal polys	3 (2%)	3 (3%)	0 (0%)	1.000
rTNSS, mean (SD)	7.8 (1.7)	7.8 (1.7)	7.7 (1.9)	.838
Rhinitis >1 year, *n* (%)^a^	123 (95%)	97 (96%)	26 (93%)	.610
Rhinitis type, *n* (%)				
Allergic rhinitis	10 (8%)	8 (8%)	2 (7%)	1.000
Nonallergic rhinitis	93 (72%)	73 (73%)	20 (71%)	.929
Mixed allergic and nonallergic	1 (1%)	1 (1%)	0 (0%)	1.000
Unknown	25 (19%)	19 (19%)	6 (21%)	.789
Prior nasal surgery, *n* (%)^b^	42 (33%)	28 (28%)	14 (50%)	.039
Medication use, *n* (%)				
Antihistamines	58 (45)	43 (43%)	—	—
Decongestant	23 (18)	6 (6%)	—	—
Oral leukotriene inhibitors	11 (8.5)	4 (4%)	—	—
Intranasal corticosteroid sprays^c^	70 (54)	32 (32%)	—	—
Intranasal anticholinergic sprays	28 (22)	16 (16%)	—	—
Immunotherapy	3 (2)	3 (3%)	—	—
Others^d^	6 (5)	4 (4%)	—	—

*Note*: Continuous variables are presented as mean (*M*) and standard deviation (SD). Categorical measures are presented as numbers (% of total).

Abbreviations: BMI, body mass index; rTNSS, 24-h reflective total nasal symptom score; SD, Standard deviation.

a Numbers and percentages represent those endorsing a “yes” response.

b Includes inferior and/or middle turbinate reduction/excision, polyp removal, septoplasty, rhinoplasty, sinuplasty, functional endoscopic sinus surgery, chemical cauterization, epistaxis control with grafting, and uvuloplasty. Some patients may have undergone multiple procedures.

c Intranasal corticosteroid sprays category includes intranasal compound sprays.

d Includes the combination of oral combination products and expectorants.

### Patient-Reported Outcome Measures

#### rTNSS

**Figure 2.**
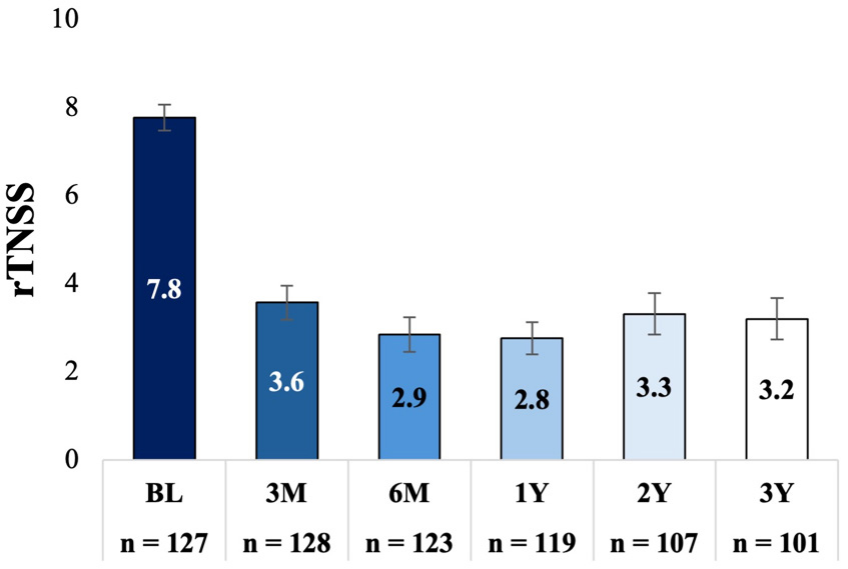
Adjusted Mean rTNSS and (95% CI) at Baseline and Each Follow-up. Bars Indicate the 95% Confidence Interval, *P* < .001, Comparing Each Follow-up Time Point to Baseline. rTNSS, 24-h Reflective Total Nasal Symptom Score.

**Figure 3.**
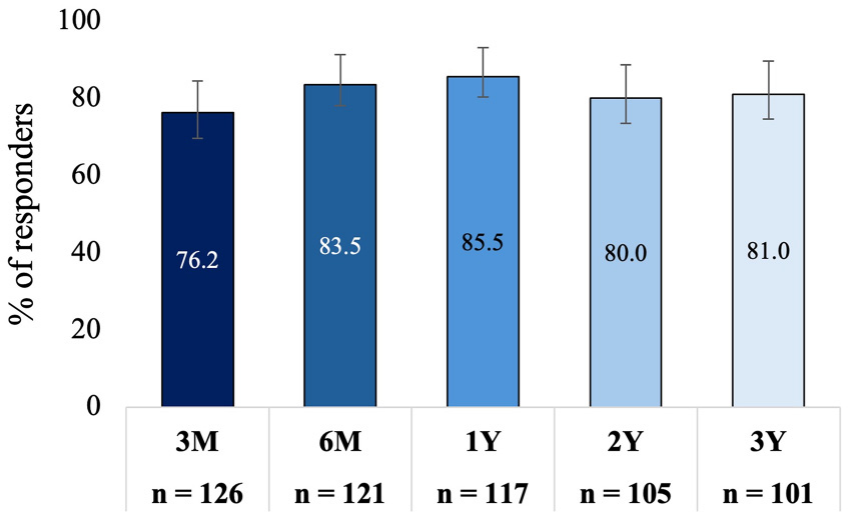
Responder Rate Over Time. A Responder was Defined as ≥30% Improvement in rTNSS From Baseline. rTNSS, 24-hour Reflective Total Nasal Symptom Score. Bars Indicate the 95% Confidence Interval.

The rTNSS mean score was significantly reduced from 7.8 (95% CI: 7.5−8.1) at baseline to 3.2 (95% CI: 2.8−3.7) at 3 years, representing a mean change of −4.5 (95% CI: −5.1 to −4.0; *P* < .001), a 58% improvement compared to baseline. Reductions in rTNSS at 3 years were consistent with those observed at 3 months (mean change: −4.2 [95% CI: −4.6 to −3.8]; *P* < .001, 54% improvement), 6 months (mean change: −5.0 [95% CI: −5.4 to −4.4]; *P* < .001, 63% improvement), 1 year (mean change: −4.9 [95% CI: −5.5 to −4.5]; *P* < .001, 64% improvement), and 2 years (mean change: −4.5 [95% CI: −5. 0 to −3.9]; *P* < .001, 58% improvement). Figure 2 shows the adjusted mean rTNSS (95% CI) at baseline and each follow-up time point. Eighty-one percent (95% CI: 72.2%−87.5%) of patients were treatment responders at 3 years. This is consistent with the percentage of responders at 3 months (76.2% [95% CI: 68.0%−82.8%]), 6 months (83.5% [95% CI: 75.8%−89.0%]), 1 year (85.5% [95% CI: 78.0%−90.7%]), and 2 years (80.8% [95% CI: 71.4%−86.5%]) (Figure 3).

The linear regression model analysis of the relationship between time (years) and the outcome measure from 1 to 3 years yielded a slope of 0.233 (Supplemental Figure 1). While the positive slope suggests a slight increase in efficacy over time; the effect did not reach statistical significance (*P* = .066). The Generalized estimating equations (GEE) model, which treated each visit as a categorical variable and compared adjacent time points, found a significant improvement from 3 to 6 months (*P* = .001) (Supplemental Table 2). A significant reduction in efficacy was observed from 12 to 24 months (*P* = .017). However, no further significant changes were observed from 24 to 36 months (*P* = .706), suggesting that efficacy remained stable in the third year.

**Figure 4.**
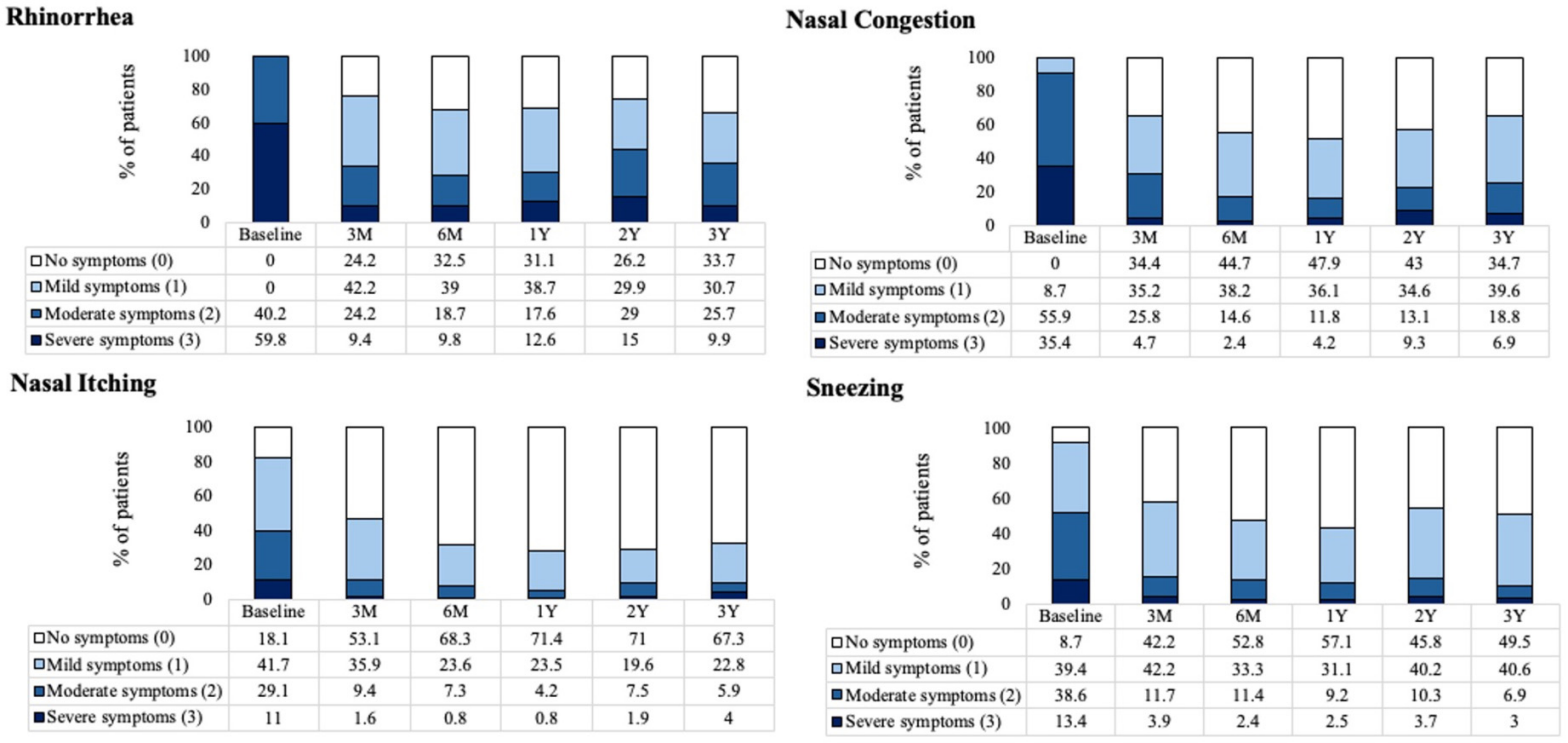
The Percentage of Patients Reporting Severity of Each rTNSS Subscore at Baseline and Follow-up. rTNSS, 24-hour Reflective Total Nasal Symptom Score.

There was a significant reduction in rTNSS individual symptom scores (rhinorrhea, congestion, nasal itching, and sneezing) at 3 years post-procedure compared to the baseline; *P* < .001 (Supplemental Figure 2). A large percentage of patients demonstrated a shift in each rTNSS severity subscore from severe/moderate at baseline to mild/none at 3 years (Figure 4). Specifically, 59.8% of patients reported severe rhinorrhea at baseline, which was reduced to 9.9% at 3 years. This corresponded to a 55.8% reduction in rhinorrhea symptoms, with mean scores improving from 2.6 at baseline to 1.2 at 3 years (Supplemental Figure 2). The percentage of patients reporting severe nasal congestion decreased from 35.4% at baseline to 6.9% at 3 years. The percentage of patients reporting severe nasal itching reduced from 11.0% at baseline to 4.0% at 3 years and patients reporting sneezing reduced from 13.4% at baseline to 3.0% at 3 years.

#### Cough and PND Symptoms

**Figure 5.**
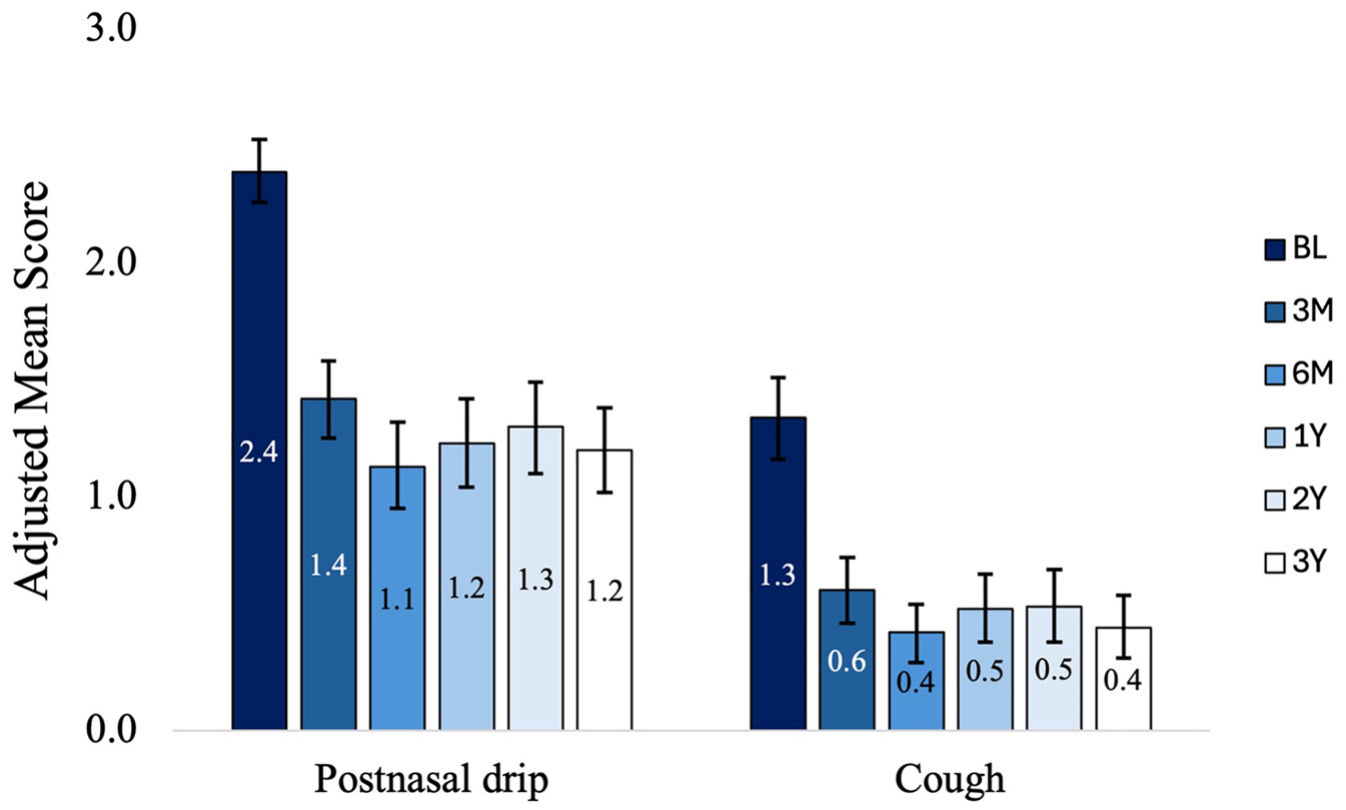
Adjusted Mean (95% CI) of PND and Cough Symptoms at Baseline and Each Follow-up. Bars Indicate the 95% Confidence Interval, *P* < .001, Comparing Each Follow-up Time Point to Baseline.

**Figure 6.**
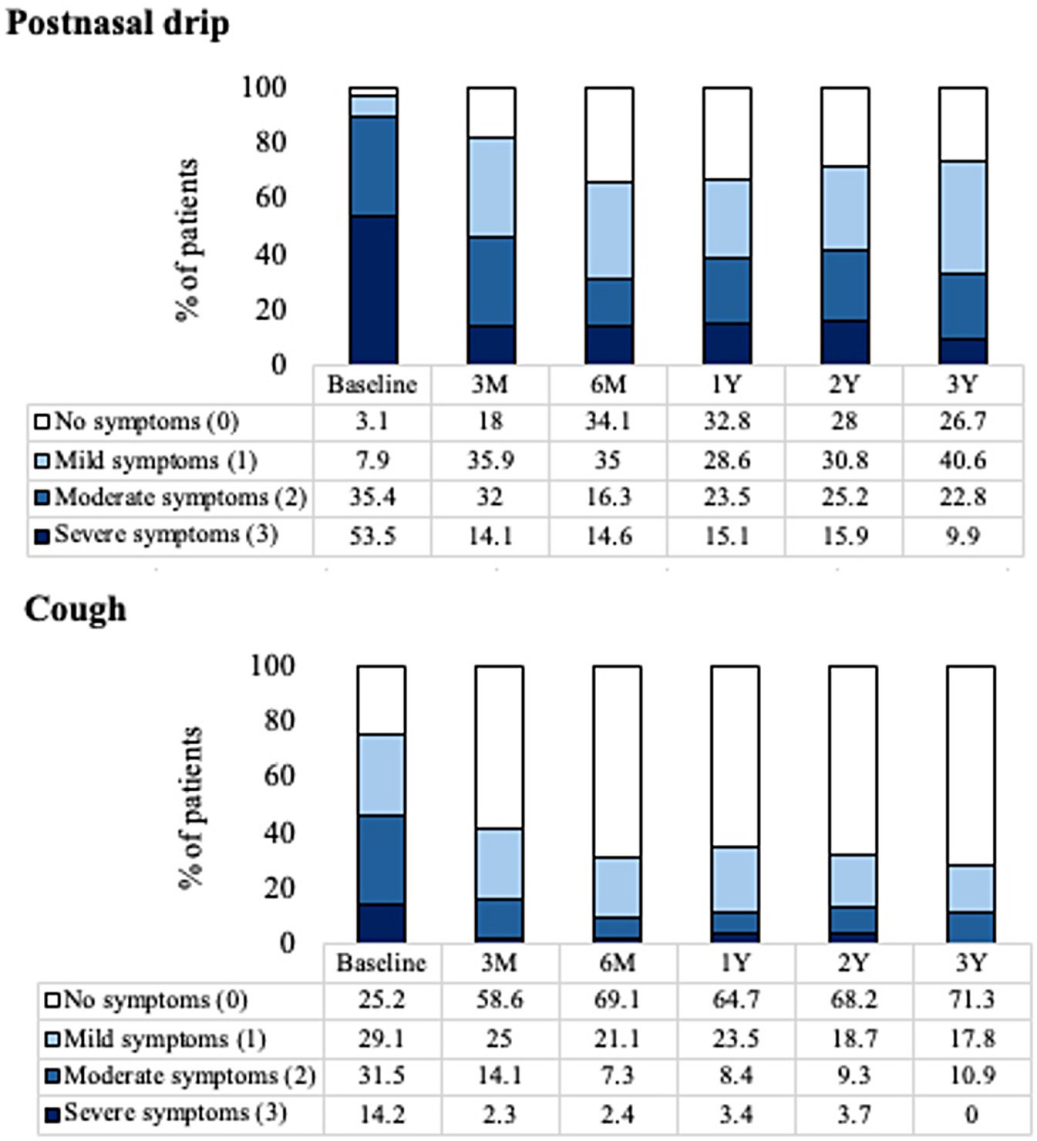
The Percentage of Patients Reporting PND and Cough Score Symptoms at Baseline and Follow-up.

Cough symptoms reduced from a mean score of 1.3 (95% CI: 1.2−1.5) at baseline to an adjusted mean score of 0.4 (95% CI: 0.3−0.6) at 3 years with a mean change of −0.9 ([95% CI: −1.1 to −0.7]; *P* < .001, a 69% reduction). PND symptoms reduced from 2.4 (95% CI: 2.3−2.5) at baseline to 1.2 (95% CI: 1.0−1.4) at 3 years with a mean change of −1.2 ([95% CI: −1.4 to −1.0]; *P* < .001, a 50% reduction). Figure 5 shows mean scores for cough and PND symptoms at baseline and each follow-up. At baseline, 14.2% and 53.5% of patients had severe cough and PND symptoms, respectively. At 3 years, based on the self-reported 3-point scale, 0.0% and 9.9% of patients had severe cough and PND symptoms, respectively. Figure 6 shows the distribution of severity of cough and PND symptoms at baseline and each follow-up time point.

### QoL

**Figure 7.**
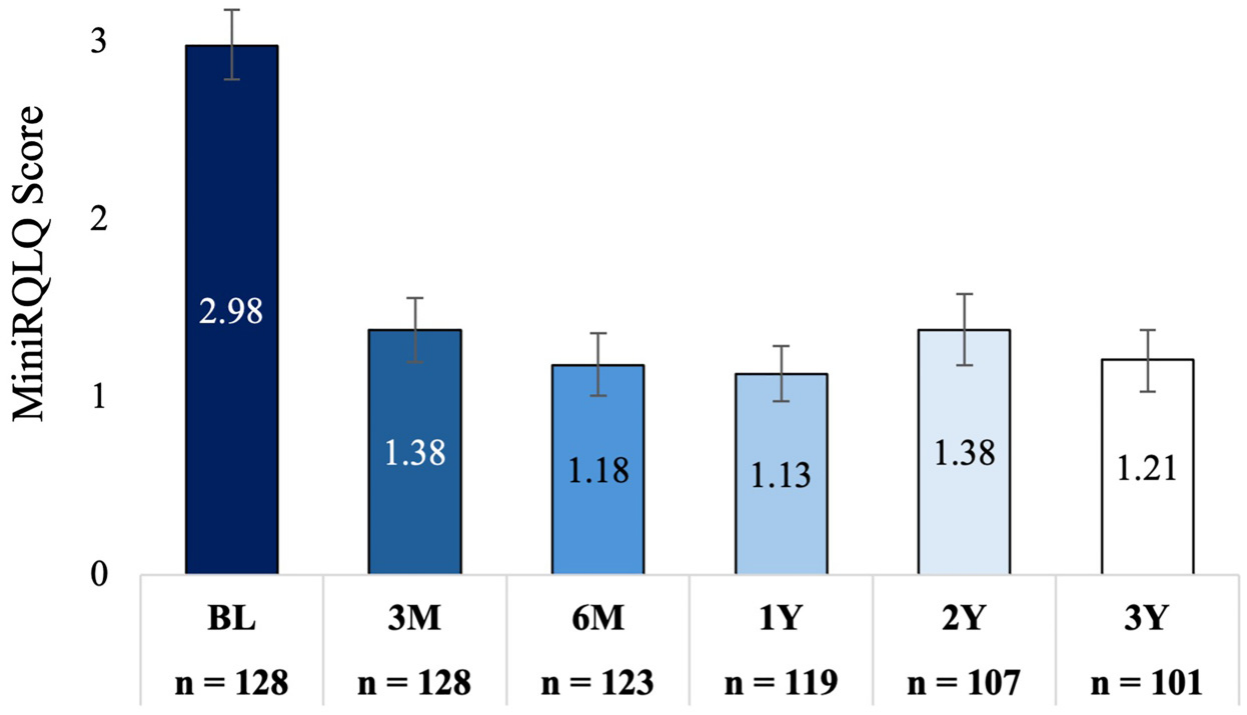
Adjusted Mean MiniRQLQ (95% CI) at Baseline and Each Follow-up. Bars Indicate the 95% Confidence Interval, *P* < .001, Comparing Each Follow-up Time Point to Baseline. MiniRQLQ, Mini Rhinoconjunctivitis Quality of Life Questionnaire.

**Figure 8.**
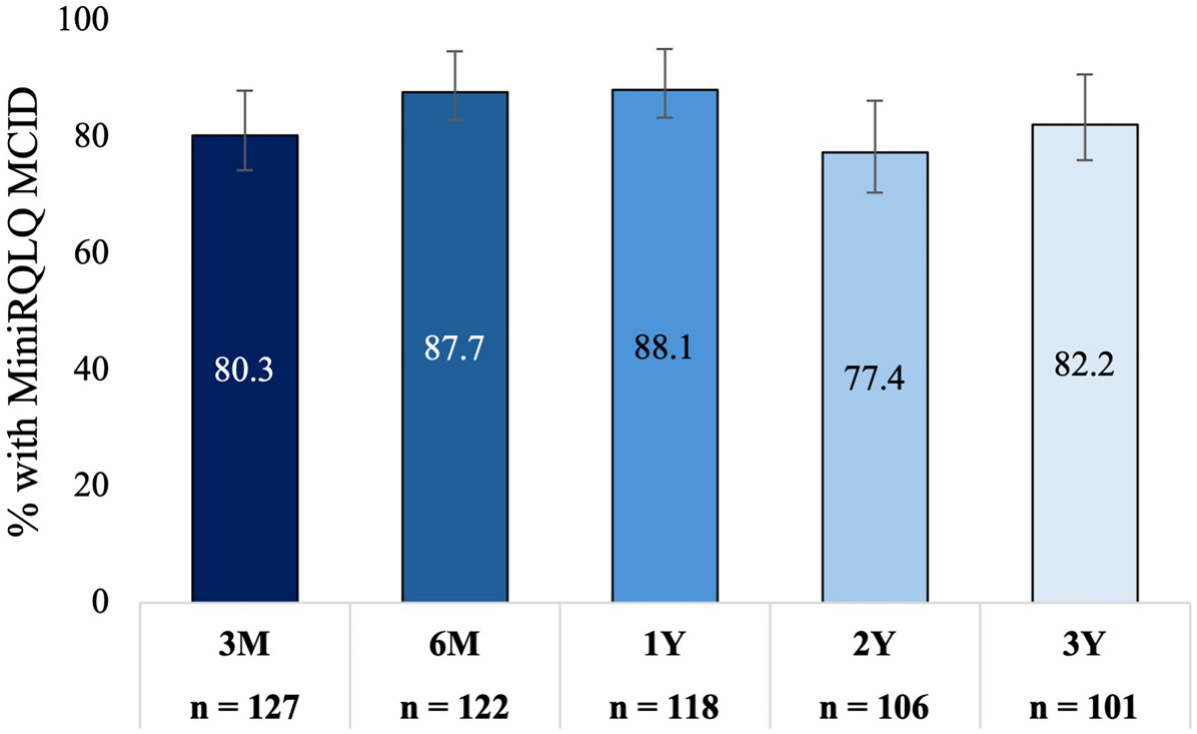
The Percentage of Patients With an MCID of ≥0.4-point Improvement in MiniRQLQ From Baseline Over Time. Bars Indicate the 95% Confidence Interval. MiniRQLQ, Mini Rhinoconjunctivitis Quality of Life Questionnaire.

MiniRQLQ score was significantly reduced from a mean of 3.0 (95% CI: 2.8−3.2) at baseline to an adjusted mean score of 1.2 (95% CI: 1.0−1.4) at 3 years; a mean change of −1.8 (95% CI: −2.0 to −1.5; *P* < .001, 60% improvement). This decrease in MiniRQLQ score is consistent with those observed at 3 months (mean change −1.6 [95% CI: −1.8 to −1.4]; *P* < .001, 53% improvement), 6 months (mean change −1.8 [95% CI: −2.0 to −1.6]; *P* < .001, 60% improvement), 1 year (mean change −1.9 [95% CI: −2.1 to −1.6]; *P* < .001, 62% improvement), and 2 years (mean change −1.6 [95% CI: −1.8 to −1.4]; *P* < .001, 53% improvement) (Figure 7). Eighty-three (82.2% [95% CI: 73.6%−88.4%]) were treatment responders at 3 years. This is consistent with the percentage of responders at 3 months (80.3% [95% CI: 72.6%−86.3%]), 6 months (87.7% [95% CI: 80.7%−92.4%]), 1 year (88.1% [95% CI: 81.1%−92.8%]), and 2 years (77.4% [95% CI: 68.5%−84.3%]) (Figure 8). All MiniRQLQ domain scores were significantly reduced at each follow-up compared to the baseline; *P* < .001 (Supplemental Table 3).

### Medications

At baseline, the most commonly used CR medications were antihistamines (45.5%) followed by intranasal corticosteroids (56.4%), anticholinergic sprays (21.8%), decongestants (14.9%), leukotriene inhibitors (6.9%), and other nasal medications (e.g., compound nasal sprays) (5%), with a few reported undergoing allergy immunotherapy (3%). At 3 years, substantial reductions (stopped or decrease in dose and/or frequency) in usage of intranasal corticosteroids (68.4%), anticholinergics (72.7%), and decongestants (80.0%) were observed with modest reductions seen for other medication classes (Table 2).

**Table 2.** Change in Medication Use.

Medication Class	Patients Using at Baseline *N* (%) (*N* = 101)	Medication Change Category	Number of Patients *N* (%)^a^	Patients Starting Medication After Baseline *N* (%)^b^
Antihistamines	46 (45.5)			8 (7.9)
		No change	22 (47.8)	
		Increased dose	5 (10.9)	
		Decreased dose	8 (17.4)	
		Stopped	11 (23.9)	
Decongestants	15 (14.9)			1 (1.0)
		No change	3 (20.0)	
		Increased dose	0 (0.0)	
		Decreased dose	2 (13.3)	
		Stopped	10 (66.7)	
Leukotriene inhibitors	7 (6.9)			0 (0.0)
		No change	3 (42.9)	
		Increased dose	0 (0.0)	
		Decreased dose	1 (14.3)	
		Stopped	3 (42.9)	
Nasal steroid sprays^c^	57 (56.4)			7 (6.9)
		No change	14 (24.6)	
		Increased dose	4 (7.0)	
		Decreased dose	7 (12.3)	
		Stopped medication	32 (56.1)	
Nasal anticholinergic Spray	22 (21.8)			7 (6.9)
		No change	6 (27.3)	
		Increased dose	0 (0.0)	
		Decreased dose	3 (13.6)	
		Stopped medication	13 (59.1)	
Immunotherapy	3 (3.0)			0 (0.0)
		No change	3 (100.0)	
		Increased dose	0 (0.0)	
		Decreased dose	0 (0.0)	
		Stopped medication	0 (0.0)	
Other^d^	5 (5.0)			1 (1.0)
		No change	2 (40.0)	
		Increased dose	0 (0.0)	
		Decreased dose	1 (20.0)	
		Stopped	2 (40.0)	

a Percentage of the patients who used the medication class at baseline.

b Percentage of the total patients.

c Nasal steroid spray includes intranasal compound spray.

d Other is the sum of oral combination products and expectorant.

### Safety

All device- and procedure-related adverse events have been previously reported through 6 months.^15^ No new device- and/or procedure-related adverse events were reported in the interval between 6 months and 3 years of follow-up. No device or procedure-related serious adverse events were reported at any time during the 3-year study period.

## Discussion

This long-term follow-up study shows that the significant improvements in CR symptoms and rhinitis-related QoL following a single TCRF treatment of PNNs were sustained through 3 years. The large percentage of treatment responders remained consistent from 3 months post-treatment through 3 years, adding to the body of knowledge that a single TCRF PNN ablation procedure is a durable and effective means of providing long-term treatment of CR symptoms.

Statistical analyses demonstrated that treatment effects were sustained through 3 years post-procedure. The GEE model, which assessed adjacent time points, showed a statistically significant improvement in efficacy from 3 to 6 months (*P* = .001), with symptom levels then stabilizing from 6 months to 12 months (*P* = .515). A modest but statistically significant reduction in efficacy was observed at 24 months compared to 12 months (*P* = .017), suggesting possible partial waning. However, no further decline was detected between 24 and 36 months (*P* = .706). While linear regression analysis showed a trend toward improved efficacy between Years 1 and 3, this was not statistically significant (*P* = .066). Together, these findings suggest initial improvement by 6 months, maintained benefit through 12 months, a slight reduction at 24 months, and overall sustained treatment effect through 36 months.

In a systematic review and meta-analysis, Yu et al. summarize the effectiveness and safety of TCRF PNN ablation for improvement of CR symptoms, including reduction in cough and PND as well as the QoL of patients with outcomes reported through 6 months.^7^ Longer-term longitudinal studies, such as this present study, are valuable for identifying possible late side effects. Long-term studies can also enable long-term cost-benefit estimates, provide information to inform policy decisions, and allow for evaluation of long-term health effects compared to alternative treatments.^19,20^

In addition to sustained reductions in symptoms of rhinorrhea, congestion, nasal itching, and sneezing, patients also reported a significant and sustained reduction in cough and PND symptoms through 3 years post-procedure. This is notable as cough and PND are common and intractable symptoms of CR, often inadequately assessed in CR studies.^2^ Prior studies of patients undergoing surgical procedures such as vidian and PNN neurectomy found that CR symptoms improved but symptoms of PND did not.^21^ One possible explanation for sustained reductions in cough symptoms following TCRF PNN ablation is reduction of nasopharyngeal mucosal hypersensitivity to physical and chemical stimulation.^22^ Gorelik et al reported a weak correlation between rhinitis symptoms and PND and cough despite an improvement in these symptoms following TCRF ablation of the PNN, suggesting that although cough and PND often coexist with CR, they might be independent of other rhinitis symptoms.^2^

A potential hypothesis to consider that might explain these findings is that denervation of the PNNs using TCRF may decrease nasal hyperreactivity and thereby reduce neuroinflammatory pathway signaling within the nasal mucosa.^23^ Although chronic cough was not objectively evaluated in this study, the durable reductions in chronic cough and PND are similar to prior studies of TCRF ablation in the PNN region.^2^ Chronic inflammation of the nasal mucosa can lead to chronic neuroinflammation of the airway.^24^ Additionally, a baseline state of nasal nerve hyperreactivity has been shown to be highly prevalent in patients with chronic inflammatory airway diseases, such as asthma, chronic cough, and chronic rhinosinusitis (with and without polyps).^25^ Further exploration of the impact of PNN ablation on chronic cough and other inflammatory airway comorbidities is worthy of additional study. Incorporation of nasal neuroinflammatory biomarkers in future studies, such as evaluating expression before and after TCRF PNN ablation in CR patients, could provide important information to improve our current understanding of how nasal nerve hyperreactivity contributes to sinonasal disease.

Vidian neurectomy and surgical PNN neurectomy have been shown to significantly reduce rhinorrhea in patients with refractory nonallergic rhinitis. Ow et al reported that cryoablation of PNN led to a 66.7% median reduction in rTNSS scores at 24 months post-procedure, with 80.7% of participants achieving clinically meaningful improvement.^8^ Studies on vidian neurectomy also demonstrated significant improvements in rhinorrhea, although direct 3-year comparative data remain limited. In contrast, TCRF treatment in the present study resulted in a 55.8% reduction in rhinorrhea symptoms over 3 years, exceeding the reported reductions for cryoablation.^26^ While vidian neurectomy has been associated with profound symptom relief, its long-term comparative efficacy relative to TCRF, and neurectomy in general, remains unclear.

This study did not specifically stratify patients by status, and no confirmatory allergy testing was performed prior to enrollment; instead, patient classification was based on self-reported history and prior physician diagnoses. Furthermore, assessment of response to an anticholinergic, such as ipratropium bromide, was not included as part of patient screening activities for enrollment. It is well recognized that CR mainly consists of allergic (AR) and nonallergic (NAR) subtypes, with some patients exhibiting mixed rhinitis.^27^ While AR is driven by an IgE-mediated immune response to allergens, NAR arises from non-immunologic triggers such as irritants, chemicals, or temperature changes.^27^ Both AR and NAR may be treated with intranasal corticosteroids and antihistamines; however, AR typically responds more robustly to these agents.^28^ In NAR, response is variable, and intranasal anticholinergics like ipratropium are often more effective for rhinorrhea.^28^ Despite these differences in medical management, interventions targeting the PNNs (eg, PNN ablation or neurectomy) have demonstrated efficacy in both AR and NAR.^26,29^

Patients in this study reported a reduction in CR medication burden at 3 years post-procedure; notably, 68% reduced or discontinued use of intranasal corticosteroids, and 73% reduced or discontinued anticholinergic nasal spray, specifically, ipratropium bromide. The reduction in CR symptoms was not driven by increased medication use; in fact, only 31.6% of patients continued or increased their use of intranasal corticosteroids, and 27.3% for anticholinergic sprays. Of the nearly half (45.5%) of patients on antihistamines at baseline, somewhat more than half (58.7%) stayed on the same dose or increased their antihistamine dose. Ongoing antihistamine use may reflect the possibility that despite a reduction in anti-inflammatory agents, patients may use antihistamines to alleviate residual allergic symptoms following TCRF treatment.

The findings reported here continue to demonstrate that TCRF treatment of the PNNs provides sustained symptom improvement (55.8% reduction in rhinorrhea at 3 years), comparable to the benefits observed with more invasive procedures. By targeting post-ganglionic nasal branches distal to the pterygopalatine ganglion, TCRF minimizes complications associated with surgical neurectomy.^5,30^ PNN neurectomy is an alternative procedure targeting the PNN and involves isolating and transecting the PNN and proximal nerve fibers without impacting the lacrimal gland but with comparable symptom relief compared to other interventions, such as vidian neurectomy.^30^ Finally, cryosurgical PNN ablation has been associated with post-procedural headaches (described as “ice cream” headaches), likely due to a trigeminal nerve response to a cold stimulus.^31^ These are not triggered by TCRF treatment. While TCRF ablation, cryotherapy, and surgical PNN neurectomy are all viable options for CR patients, the decision between an in-office procedure performed under local anesthesia versus a more invasive procedure performed under general anesthesia should be guided by patient and physician preferences.

Importantly, no serious device- or procedure-related adverse events were reported, reinforcing the favorable safety profile of TCRF as a minimally invasive approach. However, the clinical outcome assessments were self-reported and could be impacted.

The symptoms of rhinorrhea and nasal obstruction in CR are primarily due to parasympathetic nerve stimulation.^32^ By selectively ablating nerve fibers that innervate the nasal cavity, the TCRF PNN ablation procedure disrupts parasympathetic autonomic supply to the nasal mucosa. Despite potential nerve regrowth, improvements in CR symptoms remained stable through 3 years after a single TCRF session. The long-term improvements in CR symptoms observed in this study align with those reported in studies with shorter follow-up, comparing favorably to procedures such as neurectomy, cryotherapy, and laser PNN ablation.^33^ The sustained improvement in CR symptoms, reduced medication burden, and enhanced QoL observed in this study may be attributed to denervation-related downregulation of neuroinflammatory pathways.

One limitation of this study is the challenges associated with long-term follow-up studies that include patient attrition, interruption of follow-up for patients undergoing additional or alternative treatment options, and participant death.^34^ Despite these challenges, one of the strengths of this long-term follow-up study is that attrition was minimal. Additionally, this was a single arm without a placebo control. The loss of 21 patients from the study could introduce some reporting bias as there remains a possibility that those who exited the study experienced AEs or had a recurrence of CR symptoms. Furthermore, since this was a pragmatic study, medication use was not controlled. However, the findings of substantial reductions in post-treatment medication use at 3 years, particularly for intranasal corticosteroids, anticholinergic nasal sprays, and decongestants, suggest that TCRF PNN ablation offers a long-term benefit of reductions in patient medication burden.

## Conclusion

This long-term follow-up study shows that a single TCRF ablation procedure using the RhinAer device resulted in consistent and sustained improvements in CR symptoms and QoL through 3 years, along with substantial reductions in CR medication use. These findings, along with the excellent long-term safety profile, support the use of TCRF ablation of nasal nerves in the PNN region as a durable, safe, and effective method for reducing CR symptoms.

## Supplemental Material

sj-docx-1-ajr-10.1177_19458924251360889 - Supplemental material for Three-Year Outcomes After Temperature-Controlled Radiofrequency Ablation of the Posterior Nasal Nerve for Chronic Rhinitis Supplemental material, sj-docx-1-ajr-10.1177_19458924251360889 for Three-Year Outcomes After Temperature-Controlled Radiofrequency Ablation of the Posterior Nasal Nerve for Chronic Rhinitis by Jivianne T. Lee, MD, Gregory M. Abbas, MD, Daniel D. Charous, MD, Mandy Cuevas, PD, Dr. Med, Önder Göktas, Dr. Med., Patricia A. Loftus, MD, Nathan E. Nachlas, MD, Elina M. Toskala, MD, PhD, MBA, Jeremy P. Watkins, MD, and Detlef Brehmer, MD in American Journal of Rhinology & Allergy
