# Supplementary material for: Three-Year Outcomes After Temperature-Controlled Radiofrequency Ablation of the Posterior Nasal Nerve for Chronic Rhinitis
Source: Am J Rhinol Allergy. 2025 Aug 4;39(6):398–409. doi: 10.1177/19458924251360889 (PMC12480620; doi:10.1177/19458924251360889)
Supplement: sj-docx-1-ajr-10.1177_19458924251360889 - Supplemental material for Three-Year Outcomes After Temperature-Controlled Radiofrequency Ablation of the Posterior Nasal Nerve for Chronic Rhinitis [file sj-docx-1-ajr-10.1177_19458924251360889.docx]

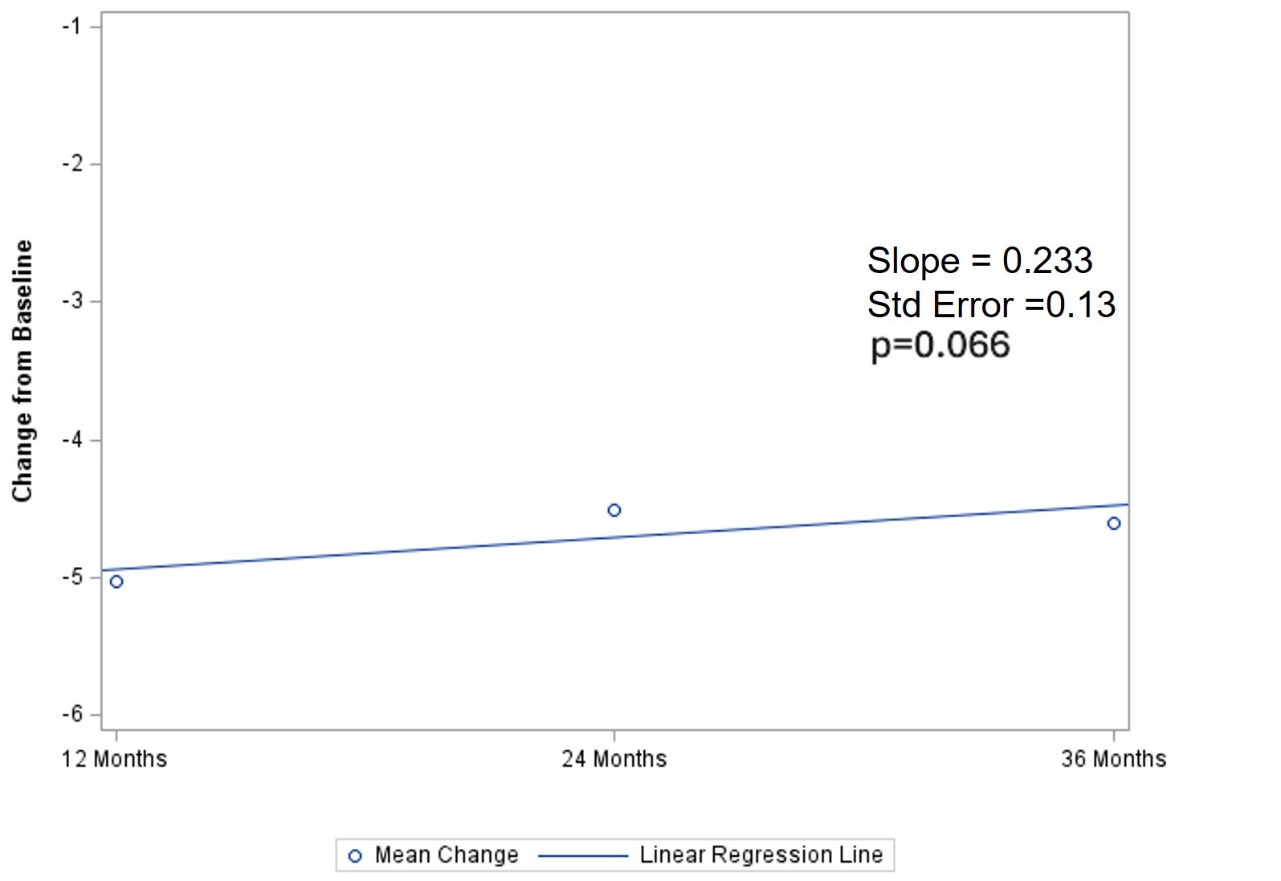


**Supplemental Figure 1.** Linear regression comparing change from baseline scores between 12 months and 36 months follow-up. The p-value represents 12 months vs. 36 months.


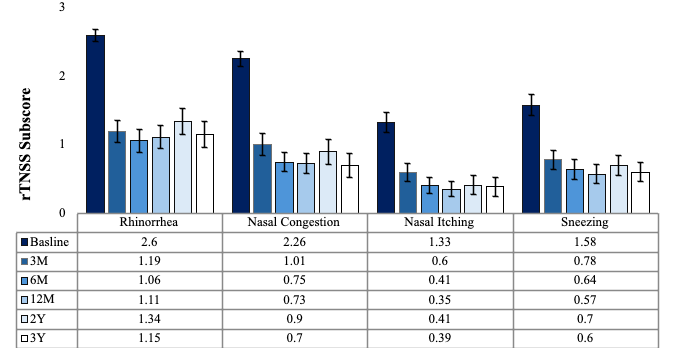


**Supplemental Figure 2.** Adjusted mean rTNSS subscores at baseline through 3 years follow-up. Bars indicate 95% confidence intervals; p<.001 comparing each follow-up to baseline for each subscore. Baseline N=127, 3 Months n= 128, 6 Months n=123, 12 Months n=119, 24 Months n=107, 36 Months n=101. rTNSS = 24-hour reflective total nasal symptom score.

**Supplemental Table 1: Study Entry Criteria**

| **Inclusion criteria**   1. Age 18 to 85 years (inclusively). 2. Willing and able to provide informed consent. 3. Willing and able to comply with the subject-specific requirements outlined in the study protocol. 4. Seeking treatment for chronic rhinitis symptoms of at least 6 months duration and willing to undergo an office-based procedure. 5. Moderate to severe symptoms of rhinorrhea (rTNSS rating of 2 or 3 for rhinorrhea). 6. Mild to severe symptoms of nasal congestion (rTNSS rating of 1, 2 or 3 for congestion). 7. rTNSS ≥ 6. |
| --- |
| **Exclusion criteria**   1. Anatomic obstructions that in the investigator’s opinion limit access to the posterior nasal passage. 2. Altered anatomy of the posterior nose as a result of prior sinus or nasal surgery or injury. 3. Active nasal or sinus infection. 4. History of significant dry eye. 5. History of any of the following: chronic epistaxis, documented episodes of significant nose bleeds in the past 3 months, rhinitis medicamentosa, head or neck irradiation. 6. Have rhinitis symptoms only on a seasonal basis due to allergies. 7. Known or suspected allergies or contraindications to the anesthetic agents and/or antibiotic medications to be used during the study procedure session. 8. Known or suspected to be pregnant or is lactating. 9. Participating in another clinical research study. 10. Has any condition that predisposes to excessive bleeding. 11. Is taking anticoagulants (e.g., warfarin, Plavix) or 325 mg aspirin that cannot be discontinued before the procedure. 12. Has previous procedure or surgery for chronic rhinitis. 13. Other medical conditions which in the opinion of the investigator would predispose the subject to poor wound healing, increased surgical risk, or poor compliance with the requirements of the study. |

**Supplemental Table 2: Percent Change in Nasal Symptom Score – Rhinorrhea**

| **Rhinorrhea** | **3 Months** | **6 Months** | **12 Months** | **24 Months** | **36 Months** |
| --- | --- | --- | --- | --- | --- |
| N | 126 | 121 | 117 | 105 | 100 |
| Mean (SD) | -55.2 (33.0) | -58.8% (37.1) | -58.1% (36.7) | -48.4% (40.7) | -56.3 (39.2) |
| Median | -50.0% | -66.7% | -66.7% | -50.0% | -50.0 |
| Q1, Q3 | -66.7%, -33.3 | -100%, -33.3% | -100, -33.3% | -100%, -33% | -100, -33.3 |
| Min, Max | -100%, 50% | -100%, 50% | -100%, 50% | -100%, 50% | -100, 50% |

Abbreviations: SD, Standard Deviation.

**Supplemental Table 3: MiniRQLQ Domain Scores at Each Follow-up Timepoint**

| **Activity limitations** | **No.^a^** | **LSM^b^** | **95% CI** | **SE** | **No.^c^** | **Mean change^b,d^** | **95% CI** | **SE** | **p-value^e^** |
| --- | --- | --- | --- | --- | --- | --- | --- | --- | --- |
| Baseline | 128 | 3.5 | 3.2 to 3.7 | 0.12 | - | - | - | - | - |
| 3 months | 128 | 1.5 | 1.3 to 1.8 | 0.12 | 127 | −1.9 | −2.2 to −1.6 | 0.15 | <.001 |
| 6 months | 123 | 1.4 | 1.1 to 1.6 | 0.13 | 122 | −2.1 | −2.4 to −1.8 | 0.16 | <.001 |
| 1 year | 119 | 1.4 | 1.2 to 1.6 | 0.11 | 118 | −2.1 | −2.4 to −1.8 | 0.15 | <.001 |
| 2 years | 107 | 1.7 | 1.4 to 2.0 | 0.14 | 106 | −1.8 | −2.1 to −1.4 | 0.16 | <.001 |
| 3 years | 101 | 1.4 | 1.2 to 1.6 | 0.12 | 101 | −2.1 | −2.4 to −1.7 | 0.16 | <.001 |
| **Practical problems** | | | | | | | | | |
| Baseline | 128 | 3.8 | 3.6 to 4.0 | 0.12 | - | - | - | - | - |
| 3 months | 128 | 1.8 | 1.6 to 2.1 | 0.12 | 127 | −2.0 | −2.2 to −1.7 | 0.14 | <.001 |
| 6 months | 123 | 1.6 | 1.4 to 1.9 | 0.12 | 122 | −2.1 | −2.5 to −1.8 | 0.16 | <.001 |
| 1 year | 119 | 1.6 | 1.4 to 1.9 | 0.13 | 118 | −2.2 | −2.5 to −1.9 | 0.15 | <.001 |
| 2 years | 107 | 1.8 | 1.6 to 2.1 | 0.14 | 106 | −1.9 | −2.3 to −1.6 | 0.16 | <.001 |
| 3 years | 101 | 1.8 | 1.5 to 2.1 | 0.14 | 101 | −2.0 | −2.3 to −1.7 | 0.16 | <.001 |
| **Nose symptoms** | | | | | | | | | |
| Baseline | 128 | 3.6 | 3.4 to 3.8 | 0.11 | - | - | - | - | - |
| 3 months | 128 | 1.6 | 1.4 to 1.8 | 0.11 | 127 | −2.0 | −2.2 to −1.7 | 0.13 | <.001 |
| 6 months | 123 | 1.3 | 1.1 to 1.5 | 0.10 | 122 | −2.2 | −2.5 to −2.0 | 0.14 | <.001 |
| 1 year | 119 | 1.3 | 1.1 to 1.5 | 0.10 | 118 | −2.3 | −2.5 to −2.0 | 0.14 | <.001 |
| 2 years | 107 | 1.7 | 1.5 to 2.0 | 0.13 | 106 | −1.9 | −2.2 to −1.6 | 0.16 | <.001 |
| 3 years | 101 | 1.5 | 1.3 to 1.7 | 0.11 | 101 | −2.1 | −2.4 to −1.8 | 0.14 | <.001 |
| **Eye symptoms** | | | | | | | | | |
| Baseline | 128 | 1.8 | 1.5 to 2.0 | 0.13 | - | - | - | - | - |
| 3 months | 128 | 0.9 | 0.7 to 1.1 | 0.09 | 127 | −0.9 | −1.1 to −0.6 | 0.14 | <.001 |
| 6 months | 123 | 0.7 | 0.5 to 0.9 | 0.09 | 122 | −1.1 | −1.4 to −0.8 | 0.14 | <.001 |
| 1 year | 119 | 0.5 | 0.4 to 0.7 | 0.07 | 118 | −1.2 | −1.5 to −1.0 | 0.13 | <.001 |
| 2 years | 107 | 0.7 | 0.5 to 0.9 | 0.10 | 106 | −1.0 | −1.3 to −0.8 | 0.14 | <.001 |
| 3 years | 101 | 0.7 | 0.5 to 0.8 | 0.10 | 101 | -1.1 | −1.4 to –0.8 | 0.15 | <.001 |
| **Other symptoms** | | | | | | | | | |
| Baseline | 128 | 2.6 | 2.3 to 2.9 | 0.14 | - | - | - | - | - |
| 3 months | 128 | 1.2 | 1.0 to 1.4 | 0.11 | 127 | −1.4 | −1.7 to −1.1 | 0.15 | <.001 |
| 6 months | 123 | 1.0 | 0.8 to 1.2 | 0.11 | 122 | −1.6 | −1.9 to −1.3 | 0.15 | <.001 |
| 1 year | 119 | 1.0 | 0.8 to 1.2 | 0.11 | 118 | −1.6 | −1.9 to −1.3 | 0.14 | <.001 |
| 2 years | 107 | 1.1 | 0.9 to 1.4 | 0.12 | 106 | −1.5 | −1.8 to −1.2 | 0.16 | <.001 |
| 3 years | 101 | 0.9 | 0.7 to 1.1 | 0.11 | 101 | -1.7 | −1.9 to −1.4 | 0.15 | <.001 |

Abbreviations: LSM, least squares mean; CI, confidence interval; SE, standard error

1. Number of patients with evaluable data.
2. Adjusted mean.
3. Number of patients with evaluable data for mean change.
4. Change in score from baseline.
5. Comparing follow-up to baseline.
